# Rediscovery of the Enigmatic *Paroxygraphis* in Xizang, China: Phylogenetic Evidence for Its Reclassification Within *Oxygraphis* (Ranunculaceae)

**DOI:** 10.1002/ece3.72306

**Published:** 2025-10-10

**Authors:** Wen‐He Li, Wan‐Ting Chen, Bo‐Wen Liu, Jia‐Min Xiao, Huan‐Yu Wu, Jian He, Lei Xie

**Affiliations:** ^1^ School of Ecology and Nature Conservation Beijing Forestry University Beijing China

**Keywords:** molecular phylogeny, morphology, new record, Ranunculeae, taxonomy

## Abstract

The enigmatic monotypic genus *Paroxygraphis* (Ranunculaceae, tribe Ranunculeae), represented solely by *P. sikkimensis* , has long posed taxonomic uncertainties due to its rarity. Through field rediscovery in southern Xizang, China, and comprehensive molecular phylogenetic analyses combining nrITS and four plastid regions, we resolved its phylogenetic position within the *Oxygraphis* clade. We found the *Oxygraphis* clade has a divergence time of about 19.26 Ma (95% HPD: 11.84–27.14 Ma) from its sister clade, and *Paroxygraphis* is nested within. Morphological and ecological characteristics, including a small herb with a rosette of basal simple leaves, longitudinally ribbed achenes, and its alpine adaptations, further support this relationship. Furthermore, the results showed that persistent sepals and unisexual flowers in Ranunculeae reflect multiple independent evolutionary origins. We propose the new combination *Oxygraphis sikkimensis* and highlight its critically endangered status due to habitat vulnerability. Lectotypification of *P. sikkimensis* is also made. This study provides new data for the floristic research of China and resolves the long‐standing taxonomic controversy of this problematic genus within Ranunculeae.

## Graphical abstract

This study resolves the long‐standing taxonomic ambiguity of the genus *Paroxygraphis* through comprehensive molecular phylogenetic analysis, demonstrating its nested position within the *Oxygraphis* clade. Based on robust phylogenetic evidence, alongside morphological and ecological similarities, we propose the reclassification of *Paroxygraphis sikkimensis* as *Oxygraphis sikkimensis*, formally designating a lectotype to stabilize the name. The research also reports the first rediscovery of this species in decades and its first confirmed record in Xizang, China, assesses it as critically endangered due to habitat vulnerability, and reveals that dioecy evolved independently within the tribe Ranunculeae.

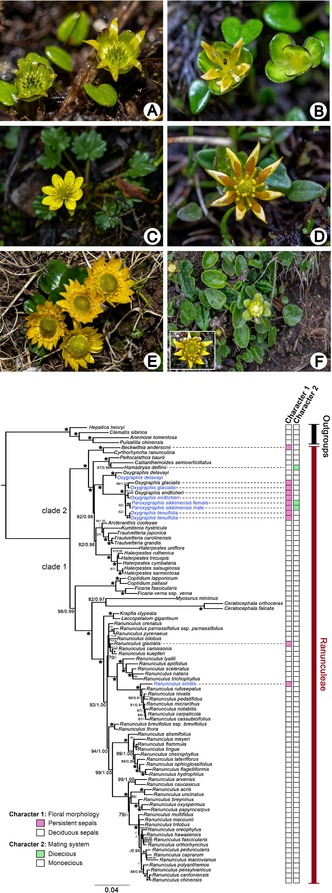

## 1 Introduction

The buttercup family, Ranunculaceae, occupies a critical phylogenetic position within the eudicot clade (Chase et al. 2016), as an early‐diverging lineage, its nearly cosmopolitan distribution across diverse ecosystems (Wang et al. 2016), and its economic importance due to ornamental and medicinal applications (Ro et al. 1997). Despite the extensive attention the family has received and the considerable amount of published studies, several genera within Ranunculaceae remain insufficiently understood and continue to pose significant taxonomic challenges.

The monotypic genus *Paroxygraphis* W. W. Smith (Ranunculaceae, tribe Ranunculeae) was first described in 1913 (Smith 1913), representing a unique taxonomic entity within the buttercup family in the south–eastern Himalayan region (India and Bhutan) (Tamura 1995). The sole species of the genus, *P. sikkimensis* W. W. Smith, is a small and inconspicuous herbaceous plant characterized by fleshy fibrous roots, simple leaves arranged in a basal rosette (with long petioles and notably small blades), and most diagnostically dioecious reproduction, a rare trait among its predominantly hermaphroditic relatives. *Paroxygraphis sikkimensis* has been recorded only in Sikkim, India and in Bhutan since its original description. This extremely limited distribution, combined with its small size, has made the species difficult to study and poorly understood.

Taxonomic studies of Ranunculaceae, particularly those by Tamura (1995) and Emadzade et al. (2010), consistently regard *Paroxygraphis* as a distinct genus within Ranunculeae. This tribe is one of the most diverse lineages within the family, containing the highest number of genera and the second‐largest number of species, only exceeded by Delphinieae. It comprises 18 recognized genera (Emadzade et al. 2010) and approximately 650 known species (Tamura 1993, 1995). Members of Ranunculeae are characterized by unitegmic ascending ovules (although *Myosurus* L. is an exception with pendent ovules) as well as petals that typically bear at least one nectary gland near the base. Although the tribe is distributed on all continents (Tamura 1995), most species occur in temperate to arctic or subantarctic regions, while in the tropics they are uncommon and largely confined to high‐altitude zones. Species in this tribe occupy diverse terrestrial and aquatic habitats, ranging from lowlands to alpine environments. Terrestrial species in particular are often adapted to extreme conditions, most commonly cold and humid settings, though some also thrive in xeric environments (Hörandl et al. 2005).

Molecular phylogenetic studies have consistently supported the monophyly of Ranunculeae (e.g., Ro et al. 1997; Wang, Lu, et al. 2009; Wang et al. 2014; Emadzade et al. 2010). Within the tribe, *Ranunculus* is the largest, globally distributed genus comprising over 600 species, whereas the remaining 17 genera are small and often narrowly distributed. These include several monotypic genera, such as *Arcteranthis*, *Beckwithia*, *Cyrtorhyncha*, *Kumlienia*, *Callianthemoides*, *Laccopetalum*, and *Peltocalathos*, that are restricted to cold temperate regions of the Americas and Eurasia. Other small genera like *Oxygraphis* and *Ceratocephala* occur mainly in the Northern Hemisphere, with some genera extending into the Southern Hemisphere (Tamura 1995; Emadzade and Hörandl 2011).

**FIGURE 1:**
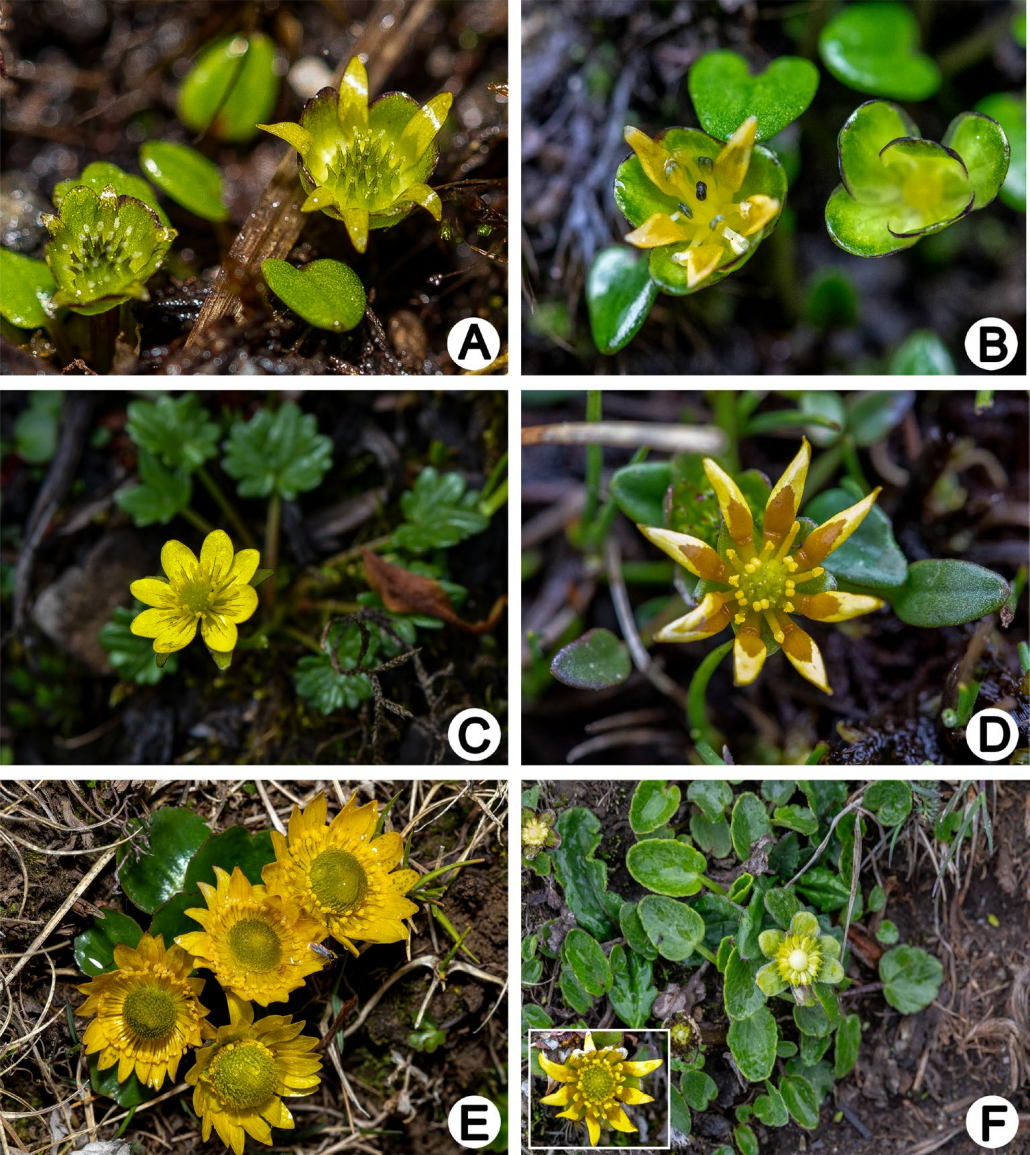
Field photographs of *Oxygraphis* and *Paroxygraphis* plants. (A, B) *Paroxygraphis sikkimensis* individuals (photographed by Bo‐Wen Liu, in Dinggyê county, Xizang); (A) Pistillate flowers; (B) Staminate flowers; (C) *Oxygraphis delavayi* in flowering stage (photographed by Lei Xie, in Bomê county, Xizang); (D) *Oxygraphis tenuifolia* in flowering stage (photographed by Bo‐Wen Liu, in Mount Sejila, Nyingchi county, Xizang); (E) *Oxygraphis glacialis* exhibiting typical morphology (photographed by Bo‐Wen Liu, in Mount Mila Pass, Maizhokunggar county, Xizang); (F) *Oxygraphis endlicheri* displaying flower, fruits, and leaves (photographed by Bo‐Wen Liu, in Latola Pass, Gyirong county, Xizang).

Based on gross morphology, distribution patterns, and ecological preferences, *P. sikkimensis* strongly suggests a close relationship with the genus *Oxygraphis* Bunge, a small genus of four or five species (Figure 1) inhabiting Asian alpine to subarctic zones (Tamura 1995; Wang et al. 2001; Rai and Rawat 2015). All species of *Oxygraphis* are acaulescent perennial herbs with fleshy fibrous roots, and their leaves are exclusively basal, simple, and petiolate. The flowers are bisexual, characterized by five persistent (rarely deciduous) sepals, multiple petals (typically 5–15), and numerous pistils (15–40). The fruits are longitudinally ribbed achenes (Tamura 1995; Wang et al. 2001). Four *Oxygraphis* species occur in China (Wang et al. 2001), and like *P. sikkimensis* , most (three or four species) retain persistent sepals post‐anthesis—a potential synapomorphy supporting their relationship. However, since persistent sepals also occur in some other taxa within Ranunculeae, such as *Beckwithia* Jeps., *Ranunculus glacialis* L., and *R. similis* Hemsl., and dioecious reproduction also occurs in *Hamadryas* Comm. ex Juss. of Ranunculeae, the systematic position of *Paroxygraphis* requires further investigation. Despite extensive molecular phylogenetic studies of Ranunculeae (Johansson 1998; Hörandl et al. 2005; Paun et al. 2005; Lehnebach et al. 2007; Hoot et al. 2008; Gehrke and Linder 2009; Hoffmann et al. 2010; Emadzade et al. 2010; Emadzade and Hörandl 2011; Wang et al. 2014), none have included *Paroxygraphis*, leaving its systematic position unresolved.

During our spring 2025 field expedition in southern Xizang, we discovered *Paroxygraphis sikkimensis* in Dinggyê County, the first documented record of this species within China. We subsequently conducted a molecular phylogenetic analysis of this species within the context of Ranunculeae. This study seeks to: (1) resolve the phylogenetic placement of *Paroxygraphis* within Ranunculeae using molecular systematics, (2) reconstruct its evolutionary history, and (3) reassess its taxonomic classification in light of phylogenetic evidence.

## 2 Materials and Methods

### 2.1 Plant Sampling and Choosing Molecular Markers

We conducted field surveys and collected leaf samples of *Paroxygraphis sikkimensis* in Dinggyê County, Xizang, China, in spring 2025. Both male and female individuals were separately collected from the field. To determine the phylogenetic position of *Paroxygraphis*, we also conducted extensive field surveys and sampling across the genus *Oxygraphis*, obtaining molecular materials from four recognized species in China for comparative analyses. Species names and delimitation of *Oxygraphis* in this study follow the treatment Flora of China (Wang et al. 2001). The recently published new species from India, *O. kumaonensis* I. D. Rai and G. S. Rawat, was not available for this study (Rai and Rawat 2015). *Ranunculus similis*, which similarly possesses a persistent calyx and was previously classified in the genus *Oxygraphis*, was also collected in the field. Besides the above eight samples (six species), we included species from all remaining Ranunculeae genera as ingroups in our molecular phylogenetic analysis. These Ranunculeae sample data were sourced from earlier studies (Wang et al. 2014).

Multiple molecular phylogenetic and phylogenomic studies (Ro et al. 1997; Wang, Lu, et al. 2009; Wang et al. 2016; Cossard et al. 2016; He et al. 2022) have shown that Anemoneae is the sister group to Ranunculeae. Therefore, this study selected species from the genera *Clematis* L., *Anemone* L., *Pulsatilla* Mill., and *Hepatica* Adans. as outgroups. In total, 83 species (representing all 18 genera) and 87 samples of Ranunculeae and four samples of Anemoneae are included in this study (Table S1). Voucher specimens of the newly generated molecular data were deposited in the Herbarium of Beijing Forestry University (BJFC, herbarium code follows Thiers 2017).

We selected a combination of molecular markers, including the nuclear ribosomal ITS region and four plastid regions (*mat*K, *psb*J‐*pet*A, *rbc*L, and *trn*L‐F) according to previously published studies (Wang et al. 2014) to cover all the genera of Ranunculeae. For newly collected samples, we obtained genome skimming data through a next‐generation sequencing method. Using the aforementioned DNA regions from GenBank as references, we assembled these five target DNA regions from the genome skimming data.

### 2.2 Genome Skimming Sequencing

Total genomic DNA was isolated from silica‐dried leaf material at Berry Genomics Co. Ltd. (https://www.bioon.com.cn/company/index/df7a2b444074) using a commercial DNA extraction kit (Tiangen Biotech Co. Ltd., Beijing, China) following the manufacturer's protocol. For library preparation, 1 μg of DNA per sample was processed with the VAHTS Universal DNA Library Prep Kit for MGI (Vazyme, Nanjing, China) to generate sequencing‐ready libraries. Paired‐end libraries of 2 × 150 bp were constructed and sequenced using a DNBSEQ‐T7 platform (BGI, Shenzhen, China). Each sequencing run produced approximately 6 Gbp of raw data per sample.

### 2.3 Phylogenetic Analyses

After assembling the five DNA regions from the newly sequenced species, sequences of each region were aligned using MAFFT v.6.833 (Katoh et al. 2005) with iterative manual optimization in Geneious v.Prime (Kearse et al. 2012), followed by the pruning of ambiguous alignment regions according to Wang et al. (2014).

Phylogenetic analyses were conducted using both maximum likelihood (ML) and Bayesian inference (BI) methods. For the ML analyses, we used IQ‐TREE v.3.0.1 (Minh et al. 2020). The best‐fit nucleotide substitution model for each dataset (nrITS, plastid, and combined) was determined using ModelFinder (Kalyaanamoorthy et al. 2017) based on the Bayesian Information Criterion (BIC). The best‐fit model was GTR+R3 for the nrITS dataset and TVM+F+R3 for the plastid dataset. For the combined dataset, a partitioned analysis was performed, with TVM+F+I+R4 selected for the plastid partition and TIM2+I+G4 for the ITS partition. Branch support for all ML analyses was assessed using 1000 ultrafast bootstrap (UFBoot) replicates (Hoang et al. 2018).

For the BI analyses, we used ExaBayes v.1.5 (Aberer et al. 2014). The GTR+GAMMA model was applied to the nrITS, plastid, and partitioned combined datasets, as is standard for nucleotide data in the software (Aberer et al. 2014). Two independent MCMC runs were conducted for 2 million generations, with trees sampled every 1000 generations. The initial 25% of trees from each run were discarded as burn‐in, and a majority‐rule consensus tree was generated to obtain posterior probabilities (PP) for branch support.

### 2.4 Molecular Dating

Due to the limited phylogenetic information provided by nrITS and plastid data, we used the combined data for molecular dating analysis according to Wang et al. (2014). Our molecular clock analysis incorporated four temporal constraints established by Emadzade and Hörandl (2011): (1) the Ranunculeae–Anemoneae divergence (46.6 Ma; Anderson et al. 2005, molecular estimate); (2) the minimum age constraint for *Myosurus* (23 Ma; Oligocene fossil evidence from Mai and Walter 1978); (3) the *R. carpaticola–R*. *notabilis* speciation event (0.725 Ma; Tomasello et al. 2020, molecular estimate); and (4) the maximum age limit for *R. caprarum* diversification (2 Ma; Stuessy et al. 2006, molecular estimate). Calibration priors were implemented according to Emadzade and Hörandl's (2011) specifications.

Divergence times were estimated using a Bayesian approach implemented in BEAST v.2.6 (Bouckaert et al. 2014). The analysis was performed using the GTR+G substitution model, an uncorrelated log‐normal relaxed molecular clock, and a Yule pure‐birth model as the tree prior. The Markov chain Monte Carlo (MCMC) analyses were run for 200 million generations, with parameters sampled every 10,000 generations. Convergence of the three independent runs was assessed in Tracer v.1.7 (Rambaut et al. 2018) by ensuring all effective sample sizes (ESS) values were above 200. After confirming convergence, the initial 25% of samples from each run were discarded as burn‐in, and the results from the runs were combined.

## 3 Results

### 3.1 Phylogeny of Ranunculeae

**FIGURE 2:**
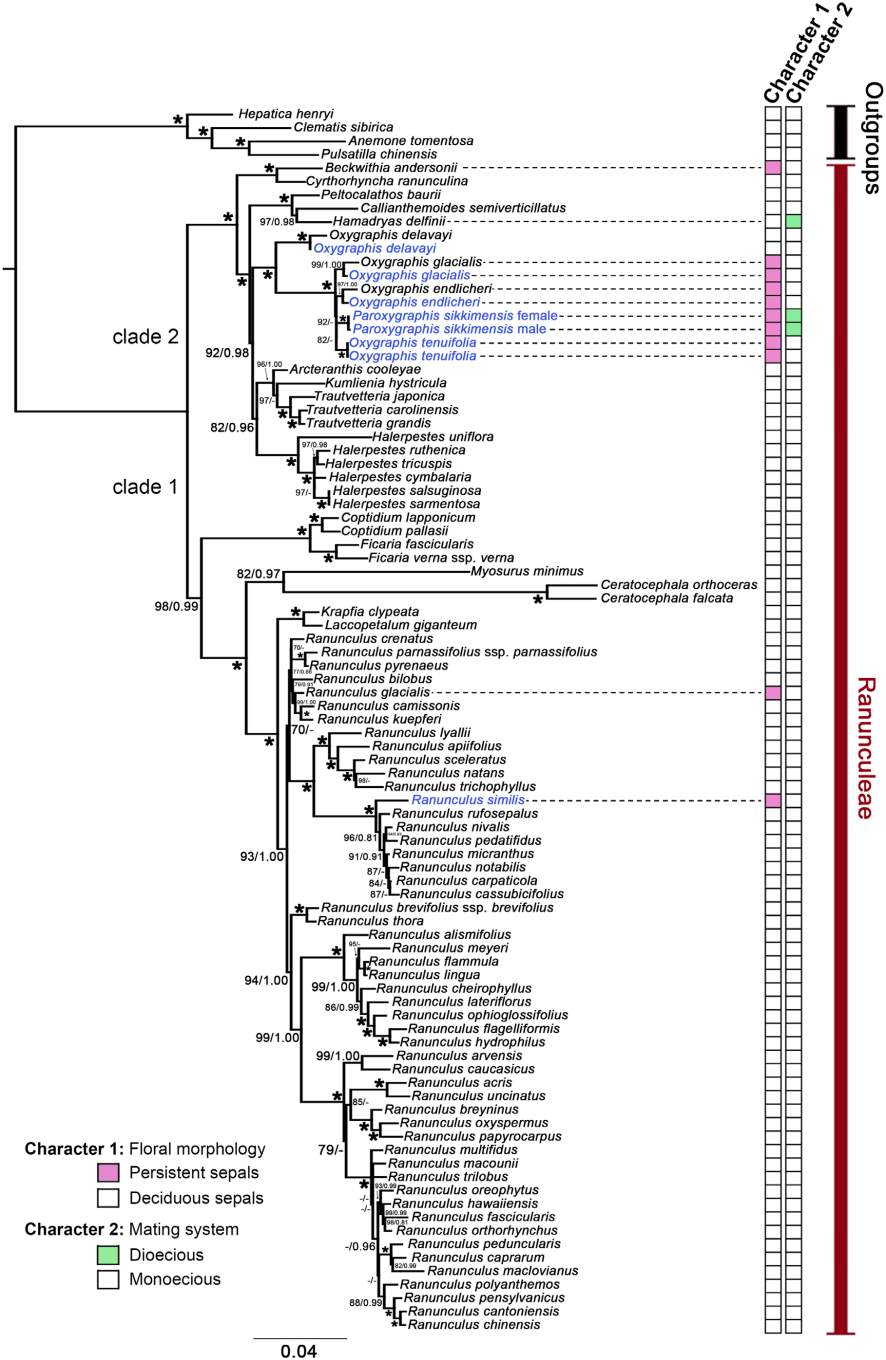
Phylogram of Ranunculeae obtained from the combined (nrITS and plastid) datasets. Numbers above and below branches are ML bootstrap (BS) percentages and Bayesian posterior probabilities, respectively. Only nodes with maximum likelihood BS > 60, and posterior probability > 0.80 were indicated. An asterisk (*) indicates maximal support (100/1.00). Taxon names in blue are newly sequenced in this study; those in black are from GenBank. The character states for floral morphology (Character 1) and mating system (Character 2) are mapped on the right.

After sites with 50% or more gaps were stripped from the alignments using the Mask Alignment function in Geneious Prime, the final aligned matrix of the concatenated plastid regions consisted of 2249 characters with 1027 variable and 743 parsimony‐informative sites. The aligned matrix of ITS sequences was 599 nucleotides in length with 343 variable and 261 parsimony‐informative sites. The ML and BI analyses yielded largely congruent tree topologies for each dataset. Contrary to the plastid topologies, the ITS topology has very low resolution, but the nodes in the ITS tree with PP > 0.95 are also recognized by plastid data (Figures S1–S4). The combined matrix included 2848 aligned characters with 1370 variable and 1004 parsimony‐informative sites. Since the combined dataset yielded the phylogeny (Figure 2) with both the highest generic‐level resolution and the strongest nodal support, discussions of intergeneric relationships in this study are predominantly based on the concatenated phylogenetic reconstruction.

When using Anemoneae species as the outgroup, all 18 sampled genera of Ranunculeae form a strongly supported monophyletic clade (nrITS data BS = 100, PP = 1.0; plastid data: BS = 100, PP = 1.0; combined data: BS = 100, PP = 1.0, Figure 2, Figures S1–S4). Our phylogenetic analyses (the plastid and the combined datasets, but not nrITS) resolved two major clades within Ranunculeae (Figure 2, Figures S1 and S2). Clade I comprised *Ranunculus* along with *Laccopetalum* Ulbr., *Krapfia* DC., *Ceratocephala* Moench, *Myosurus*, *Coptidium* (Prantl) Beurl. ex Rydb., and *Ficaria* Schaeff., while Clade II included *Trautvetteria* Fisch. et C.A. Mey., *Arcteranthis* Greene, *Kumlienia* Greene, *Halerpestes* Greene, *Oxygraphis* (containing *Paroxygraphis*), *Callianthemoides* Tamura, *Hamadryas* Tamura, *Peltocalathos* Tamura, *Cyrtorhyncha* Nutt., and *Beckwithia*. In the chloroplast gene tree (Figures S1 and S2), the *Oxygraphis* clade formed a sister‐group relationship with *Arcteranthis*, albeit with weak support (BS = 65, PP = 0.84). Combined data (Figure 2) revealed that the *Oxygraphis* clade formed a sister relationship with a branch comprising *Kumlienia*, *Arcteranthis*, *Halerpestes*, and *Trautvetteria* (BS = 92, PP = 0.98).

The nrITS sequences failed to resolve the *Oxygraphis* clade (Figures S3 and S4). *Oxygraphis delavayi* Franch. (the only *Oxygraphis* species without persistent sepals) was separated from the other species of *Oxygraphis*, but with low overall support. However, *Paroxygraphis* formed a strongly supported clade with all the other *Oxygraphis* species in the nrITS tree (BS = 100, PP = 1). In this clade, all the species have persistent sepals.

The plastid and combined datasets consistently placed both male and female individuals of *P. sikkimensis* within the *Oxygraphis* clade (Figure 2 Figures S1 and S2, combined data BS = 100, PP = 1). Within this clade, *O. delavayi* represents the earliest‐diverging lineage and is uniquely characterized by its lack of persistent sepals, a morphological character retained in all other congeneric species as well as *P. sikkimensis* . Within the clade of persistent‐sepal *Oxygraphis* species (Figure 2, combined data BS = 100, PP = 1), the relationships among *O. endlicheri*, *O. tenuifolia*, *O. glacialis*, and *Paroxygraphis* remain unresolved.

Within Ranunculeae, the other dioecious genus *Hamadryas* forms a sister‐group relationship with *Callianthemoides* (BS = 97, PP = 0.98, Figure 2), while showing no direct ancestral connection with *Paroxygraphis*. *Beckwithia*, another genus characterized by persistent sepals, forms a sister‐group relationship with *Cyrtorhyncha* (BS = 100, PP = 1.0, Figure 2), also showing no direct connection to *Paroxygraphis*.

### 3.2 Molecular Dating

**FIGURE 3:**
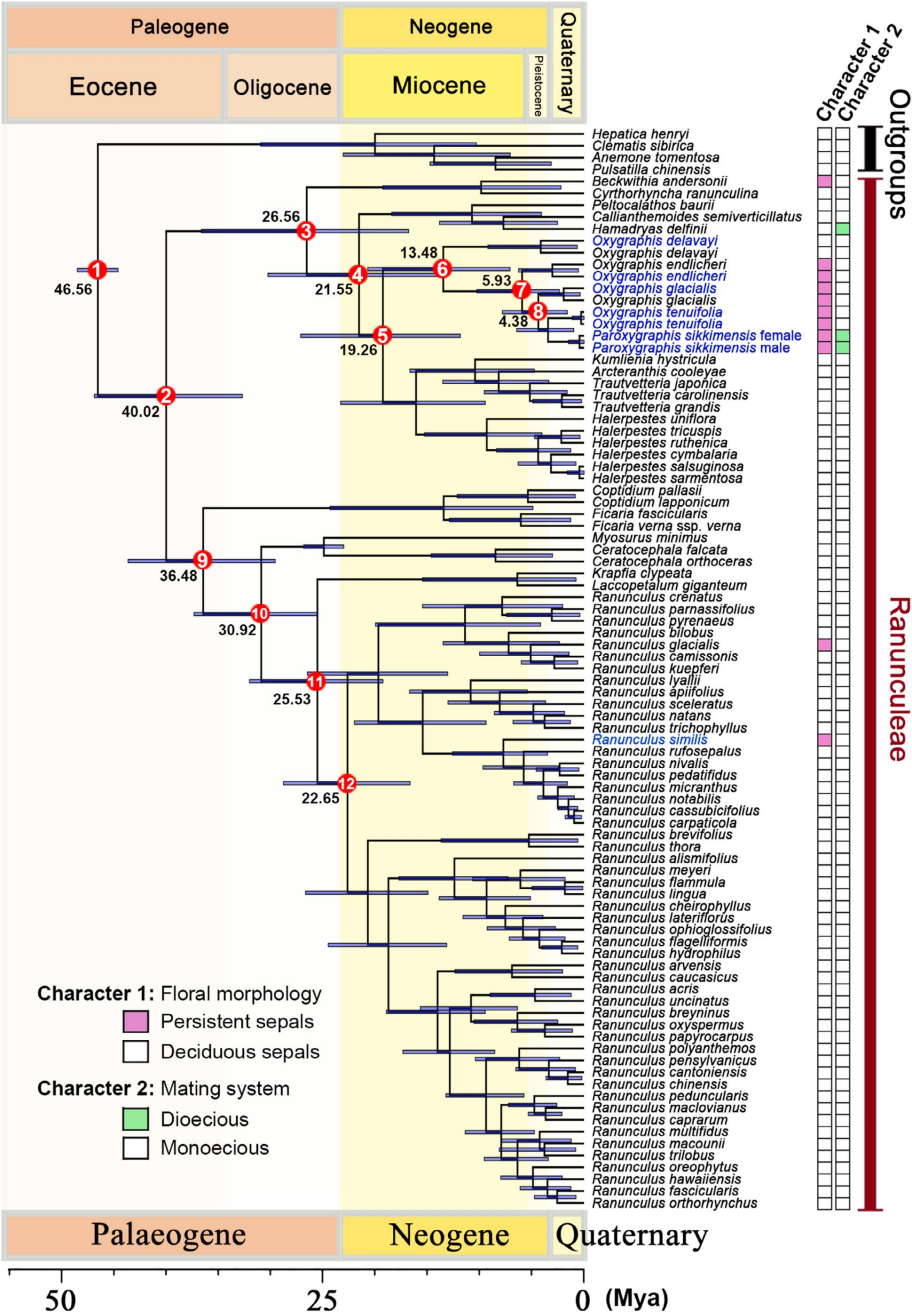
Maximum clade credibility chronogram of Ranunculeae obtained from the combined (nrITS and plastid) datasets. Blue bars on the nodes represent the 95% highest posterior density (HPD) intervals for the age estimates. Taxon names in blue are newly sequenced in this study, and those in black are retrieved from GenBank. The character states for floral morphology (Character 1) and mating system (Character 2) are mapped on the right. The geological time scale (Gradstein et al. 2004) is shown at the bottom.

The analyses based on the combined dataset resulted in very similar divergence‐time estimates (Figure 3, Table 1) for genera of Ranunculeae with previous studies (Emadzade et al. 2010; Wang et al. 2014). Our time estimates suggest that extant Ranunculeae date to the Eocene (node 1, 46.56 Ma) (95% highest posterior density, HPD: 44.63–48.52 Ma). The generic diversification of clade I occurred in the Late Eocene (node 9, 36.48 Ma, 95% HPD: 29.58–43.67 Ma). The largest genus in Ranunculeae, *Ranunculus*, diversified (crown age) at 25.53 Ma (node 11, 95% HPD: 19.25–32.04 Ma). The age of crown clade II is estimated to be 24.95 Ma (node 3, 95% HPD: 17.21–33.17 Ma). The *Oxygraphis* clade diverged from its close relatives at 19.26 Ma (node 5, 95% HPD: 11.84–27.14 Ma), and the current species of this clade diversified at 13.48 Ma (node 6, 95% HPD: 7.09–20.71 Ma). In the *Oxygraphis* clade, the crown group age of the subclade containing species with persistent sepals is 5.93 Ma (node 7, 95% HPD: 2.36–10.26 Ma). *Paroxygraphis sikkimensis* split from its sister clade at 4.38 Ma (node 8, 95% HPD: 1.60–7.83 Ma).

**TABLE 1:** Divergence time estimates (Ma) for Ranunculeae. The node numbers are shown in Figure 3.

Nodes	Mean height (Ma)	Median height (Ma)	Lower 95% HPD (Ma)	Upper 95% HPD (Ma)	Posterior probability
1	46.56	46.56	44.63	48.52	1.00
2	40.02	40.24	32.72	46.90	1.00
3	26.56	26.34	16.80	36.66	1.00
4	21.55	21.23	13.45	30.27	1.00
5	19.26	18.92	11.84	27.14	0.94
6	13.48	13.10	7.09	20.71	1.00
7	5.93	5.54	2.36	10.26	1.00
8	4.38	4.06	1.60	7.83	0.96
9	36.48	36.34	29.58	43.67	0.96
10	30.92	30.49	25.54	37.35	1.00
11	25.53	25.41	19.25	32.04	1.00
12	22.65	22.54	16.66	28.78	1.00

## 4 Discussion

### 4.1 Phylogenetic Relationships Within Ranunculeae

Our plastid and combined datasets recovered the two major clades (Figures 2 and 3) in Ranunculeae as reported in earlier studies (Emadzade et al. 2010; Emadzade and Hörandl 2011; Wang et al. 2014). Within Clade II, however, phylogenetic relationships remained partially unresolved. While the *Oxygraphis* clade was consistently placed in Clade II, its closest relatives were unclear: the combined data weakly suggested a sister relationship to a clade containing *Kumlienia*, *Arcteranthis*, *Trautvetteria*, and *Halerpestes*, whereas plastid data alone weakly supported *Arcteranthis* as sister (Figures S1 and S2; BS = 65, PP = 0.84). By contrast, deeper divergences in Clade I allowed for clearer inference of intergeneric relationships.

The unresolved intergeneric relationships within Clade II likely stem from the insufficient phylogenetic signal provided by limited Sanger sequencing data, which may be inadequate for disentangling recent rapid radiations. In contrast, genomic‐scale data have demonstrated strong potential to significantly enhance phylogenetic resolution, as evidenced by studies utilizing whole plastid genomes (e.g., Song et al. 2024 in Nymphaeales) and nuclear genomic data (e.g., Tomasello et al. 2020 in *Ranunculus*). In Ranunculaceae, widespread cytonuclear discordance further complicates phylogenetic reconstruction (He et al. 2021, 2022), suggesting that analyses based solely on combined nrITS and a few plastid markers may yield unreliable evolutionary histories. In such cases, nuclear genomic data are often better reflect the true species relationships (Liu et al. 2022; Lyu et al. 2023). Therefore, this study emphasizes the importance of employing genomic approaches, particularly nuclear genome data, in future efforts to reconstruct the phylogeny of the Ranunculeae and to clarify the problematic intergeneric relationships within Clade II.

### 4.2 Origin and Evolution of *Oxygraphis* and *Paroxygraphis*

*Oxygraphis* is a genus within clade II of Ranunculeae that exhibits special adaptations to alpine and arctic environments. All four or five species in this genus can be found in the Hengduan Mountains, the Qinghai–Xizang Plateau, and the Himalayan regions, with three or four of them being endemic to this region (Wang et al. 2001; Rai and Rawat 2015). Only *O. glacialis* extends further into the cold regions of northern Asia, with its range reaching as far as Alaska in North America. Although polyploidization is prevalent in many taxa within Ranunculeae (Hörandl et al. 2005; Paun et al. 2005, 2006; Baltisberger and Widmer 2009; Cires et al. 2013), all currently reported *Oxygraphis* species are diploid (2*n* = 16, *x* = 8, Yang 2000).

Phylogenetic analyses confirm that *O. delavayi* , the only species in the genus with deciduous sepals, represents the earliest diverging lineage within *Oxygraphis* (Wang et al. 2014), having split from the persistent‐sepal clade during the Miocene (13.48 Ma, 95% HPD: 7.09–20.71 Ma, Figures 2 and 3, Table 1). The remaining species, including *Paroxygraphis sikkimensis* (which bears only 5–6 petals, notably fewer than other congeners; Tamura 1995; Figure 1), form a clade characterized by persistent sepals as a synapomorphy. This trait unites *O. glacialis* , *O. tenuifolia* , *O. endlicheri*, and *P. sikkimensis* in our phylogenetic reconstructions, even in the nrITS tree (Figures S3 and S4). The persistent‐sepal clade diversified in the late Miocene (5.93 Ma, 95% HPD: 2.36–10.26 Ma, Figures 2 and 3, Table 1), a period marked by severe cooling in Asia that may have favored the retention of sepals as a protective adaptation for developing fruits. Beyond *Oxygraphis*, persistent sepals have evolved independently in several other Ranunculeae lineages (e.g., *Beckwithia*, *Ranunculus glacialis* , and *R. similis* ; Figures 2 and 3). As noted by Emadzade et al. (2010), these taxa predominantly inhabit high alpine or Arctic environments, suggesting that persistent sepals represent a homoplastic trait associated with protection from wind and extreme cold.

Diversification of *Oxgraphis* also occurred within the broader biogeographic context of the Qinghai‐Tibetan Plateau (QTP), which hosts one of Earth's most remarkable and recently assembled alpine floras (Wen et al. 2014). The region's complex uplift history, especially from the late Miocene onward, created a patchwork of high‐elevation habitats (Spicer et al. 2003; Guo et al. 2008). Coupled with intense cooling during the Pliocene and subsequent Quaternary glacial–interglacial cycles, these processes formed a dynamic “species pump” that promoted rapid diversification across many plant groups (Wen et al. 2013). The nearly simultaneous radiations observed not only in *Oxygraphis* but also in genera such as *Pedicularis* (Eaton and Ree 2013), *Saussurea* (Wang, Susanna, et al. 2009), *Rhodiola* (Zhang et al. 2014), and *Gentiana* (Zhang et al. 2009) highlight the pivotal role of the QTP's Plio–Quaternary environmental upheavals in shaping the composition and evolutionary trajectories of Asia's alpine flora.

The results of this study indicate that *Paroxygraphis* is embedded within the *Oxygraphis* clade, with an estimated divergence time from its closest sister group of approximately 4.38 Ma (95% HPD: 1.60–7.83 Ma). *Paroxygraphis sikkimensis* is also an alpine and cold‐adapted species, restricted to high‐elevation zones of the southeastern Himalayas. Unlike most *Oxygraphis* species inhabiting alpine meadows, *P. sikkimensis* grows exclusively on moss‐covered, damp rock crevices at elevations of about 3800 m. Due to its diminutive size, early anthesis (May), and special habitat, this species is exceptionally hard to find and easily overlooked in field surveys.

While *Paroxygraphis* is morphologically highly similar to *Oxygraphis*, traditional classifications have consistently treated it as a distinct genus based primarily on its dioecious reproductive system (Smith 1913; Tamura 1995; Emadzade et al. 2010). The evolution and maintenance of dioecy, despite its demographic costs, must be driven by substantial fitness advantages. Two primary selective forces, inbreeding avoidance and sexual specialization (Charlesworth and Charlesworth 1978; Bawa 1980; Case and Barrett 2004), are hypothesized to provide this advantage. Although dioecy is central to most animals, it is relatively rare in angiosperms, occurring in only about 6%–7% of species (Negrutiu et al. 2001). However, this rarity belies a broad phylogenetic distribution, as dioecy is found in approximately 40%–50% of all angiosperm families (Montalvão et al. 2021). This pattern indicates that dioecy is not merely a historical artifact but has arisen independently on numerous occasions throughout angiosperm evolution.

Within Ranunculeae, the South American genus *Hamadryas* similarly exhibits a dioecious sexual system (Tamura 1995; Hoot et al. 2008). Our molecular phylogenetic results demonstrate that *Hamadryas* and *Callianthemoides* (Figures 2 and 3) form a sister group, sharing no direct common ancestry with *Paroxygraphis*. This indicates that dioecy has evolved independently at least two times within Ranunculeae. Moreover, certain genera within Ranunculaceae, such as *Clematis* L. and *Thalictrum* L., contain both hermaphroditic and dioecious species, demonstrating sexual system variability within these genera (Tamura 1995). Considering the robust molecular phylogenetic relationships revealed by our analyses, we recommend recognizing *Paroxygraphis* as congeneric with *Oxygraphis*.

## 5 Taxonomic Treatment

Oxygraphis sikkimensis (W. W. Smith) Wen He Li & L. Xie, comb. nov.

*Basionym*: *Paroxygraphis sikkimensis* W. W. Smith, Rec. Bot. Surv. India 4: 344, 1913.

### 5.1 Type

*Type*: India, Sikkim, near Changu bungalow, in the Dikchu valley, in the Chola Range below the Tanka La, in the wetter ranges at 12000–14000 ft., *Smith 3204* [lectotype here designated: K (K000692711!); isolecto E!]

*Typification notes*: At the time of its publication (Smith 1913), *Paroxygraphis sikkimensis* was described with reference to multiple herbarium specimen gatherings but without explicit designation of a holotype. These specimens collectively constitute syntypes under Articles 9.5 and 40.2 of the International Code of Nomenclature (Turland et al. 2018). Herein, we formally designate specimen *Smith 3204* (K000692711) at the Kew Herbarium as the lectotype to stabilize the taxonomic application of this name. The selected lectotype best represents the species' diagnostic characters as described in the protologue and shows optimal preservation of key morphological features.

*Additional specimens examined*: China, Xizang, Dinggyê County, near the China‐Nepal border (Due to the species' extremely limited distribution and conservation concerns, precise geographic coordinates have been intentionally omitted from this publication to prevent anthropogenic disturbances.), alt. 3850 m, 18 May 2025, *Wen‐He Li & Bo‐Wen Liu 2025051806* (BJFC); alt. 3800 m, 19 May 2025, *Wen‐He Li & Bo‐Wen Liu 2025051905* (BJFC).

*Ecology, distribution, and status*: This species was previously known only from a few scattered localities in India (Sikkim) and Nepal (Smith 1913). We hereby report its first occurrence in China, from the southern Xizang Autonomous Region. In Dinggyê County, the plants were found exclusively growing within moss cushions, primarily species of *Pogonatum* P. Beauv. and *Dicranum* Hedw., on moist limestone cliff faces at an elevation of approximately 3800 m. This suggests a narrow ecological preference for humid, alpine microhabitats associated with calcareous rock substrates. The local population is restricted to a small area, supporting only a few hundred individuals. The surrounding habitat consists of high‐altitude alpine meadows and rocky slopes, with the moss‐covered limestone outcrops providing critical microrefugia that maintain high humidity and shelter from wind and insolation. No other obligate calciphilous mosses or vascular plants were recorded in immediate association, though common alpine shrubs and forbs occur scattered across the adjacent slopes. This species' habitat is highly vulnerable to anthropogenic disturbances, which may lead to population degradation or even local extinction. According to the IUCN red list categories and criteria (IUCN Standards and Petitions Committee 2022), *Oxygraphis sikkimensis* should be categorized as critically endangered (CR).

## 6 Conclusions

Our integrative study resolves the century‐old taxonomic ambiguity surrounding *Paroxygraphis* by demonstrating its nested position within *Oxygraphis*, supported by molecular phylogenetics, morphological synapomorphies, and biogeographic evidence. The rediscovery of *O. sikkimensis* in Xizang expands its known range and underscores the Himalayan region as a hotspot for alpine plant diversification. Convergent evolution of persistent sepals and dioecy in Ranunculeae highlights adaptive responses to harsh environments. Taxonomic reclassification of *Paroxygraphis* under *Oxygraphis* rationalizes its phylogenetic affinity while preserving diagnostic traits. However, the species' extreme habitat specialization and limited distribution necessitate urgent conservation measures. This work not only advances systematic understanding of Ranunculeae but also emphasizes the importance of combining field exploration with molecular tools to unravel cryptic biodiversity in alpine ecosystems. The discovery of *Oxygraphis sikkimensis* further suggests that our understanding of the flora in China's Himalayan regions remains incomplete.

## Author Contributions

**Wen‐He Li:** data curation (equal), formal analysis (equal), methodology (equal), resources (lead), software (equal), validation (lead), visualization (lead), writing – original draft (equal), writing – review and editing (equal). **Wan‐Ting Chen:** data curation (lead), resources (lead), writing – review and editing (equal). **Bo‐Wen Liu:** data curation (equal), methodology (equal), software (supporting), visualization (supporting), writing – original draft (supporting), writing – review and editing (supporting). **Jia‐Min Xiao:** data curation (equal), investigation (equal), resources (equal), supervision (supporting), visualization (equal). **Huan‐Yu Wu:** data curation (supporting), investigation (supporting), resources (supporting), supervision (supporting), visualization (equal). **Jian He:** conceptualization (lead), funding acquisition (equal), project administration (lead), supervision (lead), writing – review and editing (lead). **Lei Xie:** conceptualization (equal), funding acquisition (lead), project administration (lead), supervision (lead), writing – review and editing (equal).

## Conflicts of Interest

The authors declare no conflicts of interest.

## Supporting information


**Figure S1:** Maximum likelihood (ML) phylogram of Ranunculeae, inferred using IQ‐TREE from the plastid dataset under the TVM+F+R3 model. Numbers at the nodes represent ML bootstrap (BS). Taxon names in blue indicate species newly sequenced for this study, while those in black are from sequences retrieved from GenBank.
**Figure S2:** Bayesian inference (BI) phylogram of Ranunculeae, inferred from the plastid dataset using ExaBayes under the GTR+GAMMA model. Numbers at the nodes represent Bayesian posterior probabilities (PP). Taxon names in blue indicate species newly sequenced for this study, while those in black are from sequences retrieved from GenBank.
**Figure S3:** Maximum likelihood (ML) phylogram of Ranunculeae, inferred using IQ‐TREE from the ITS dataset under the GTR+R3 model. Numbers at the nodes represent ML bootstrap (BS). Taxon names in blue indicate species newly sequenced for this study, while those in black are from sequences retrieved from GenBank.
**Figure S4:** Bayesian inference (BI) phylogram of Ranunculeae, inferred from the ITS dataset using ExaBayes under the GTR+GAMMA model. Numbers at the nodes represent Bayesian posterior probabilities (PP). Taxon names in blue indicate species newly sequenced for this study, while those in black are from sequences retrieved from GenBank.

**Table S1:** Species, GenBank accession numbers, and vouchers of the sequences used in this study.

## Data Availability

The DNA sequences generated in the present study have been deposited in the National Center for Biotechnology Information (NCBI) database. The accession numbers and the information on the voucher specimens are available in Table S1. The voucher specimens have been deposited at BJFC.
