# Supplementary material for: Rediscovery of the Enigmatic Paroxygraphis in Xizang, China: Phylogenetic Evidence for Its Reclassification Within Oxygraphis (Ranunculaceae)
Source: Ecol Evol. 2025 Oct 10;15(10):e72306. doi: 10.1002/ece3.72306 (PMC12513732; doi:10.1002/ece3.72306)
Supplement: Supplementary file 1 — Figure S1: Maximum likelihood (ML) phylogram of Ranunculeae, inferred using IQ‐TREE from the plastid dataset under the TVM+F+R3 model. Numbers at the nodes represent ML bootstrap (BS). Taxon names in blue indicate species newly sequenced for this study, while those in black are from sequences retrieved from GenBank. Figure S2: Bayesian inference (BI) phylogram of Ranunculeae, inferred from the plastid dataset using ExaBayes under the GTR+GAMMA model. Numbers at the nodes represent Bayesian posterior probabilities (PP). Taxon names in blue indicate species newly sequenced for this study, while those in black are from sequences retrieved from GenBank. Figure S3: Maximum likelihood (ML) phylogram of Ranunculeae, inferred using IQ‐TREE from the ITS dataset under the GTR+R3 model. Numbers at the nodes represent ML bootstrap (BS). Taxon names in blue indicate species newly sequenced for this study, while those in black are from sequences retrieved from GenBank. Figure S4: Bayesian inference (BI) phylogram of Ranunculeae, inferred from the ITS dataset using ExaBayes under the GTR+GAMMA model. Numbers at the nodes represent Bayesian posterior probabilities (PP). Taxon names in blue indicate species newly sequenced for this study, while those in black are from sequences retrieved from GenBank. [file ECE3-15-e72306-s001.pdf]

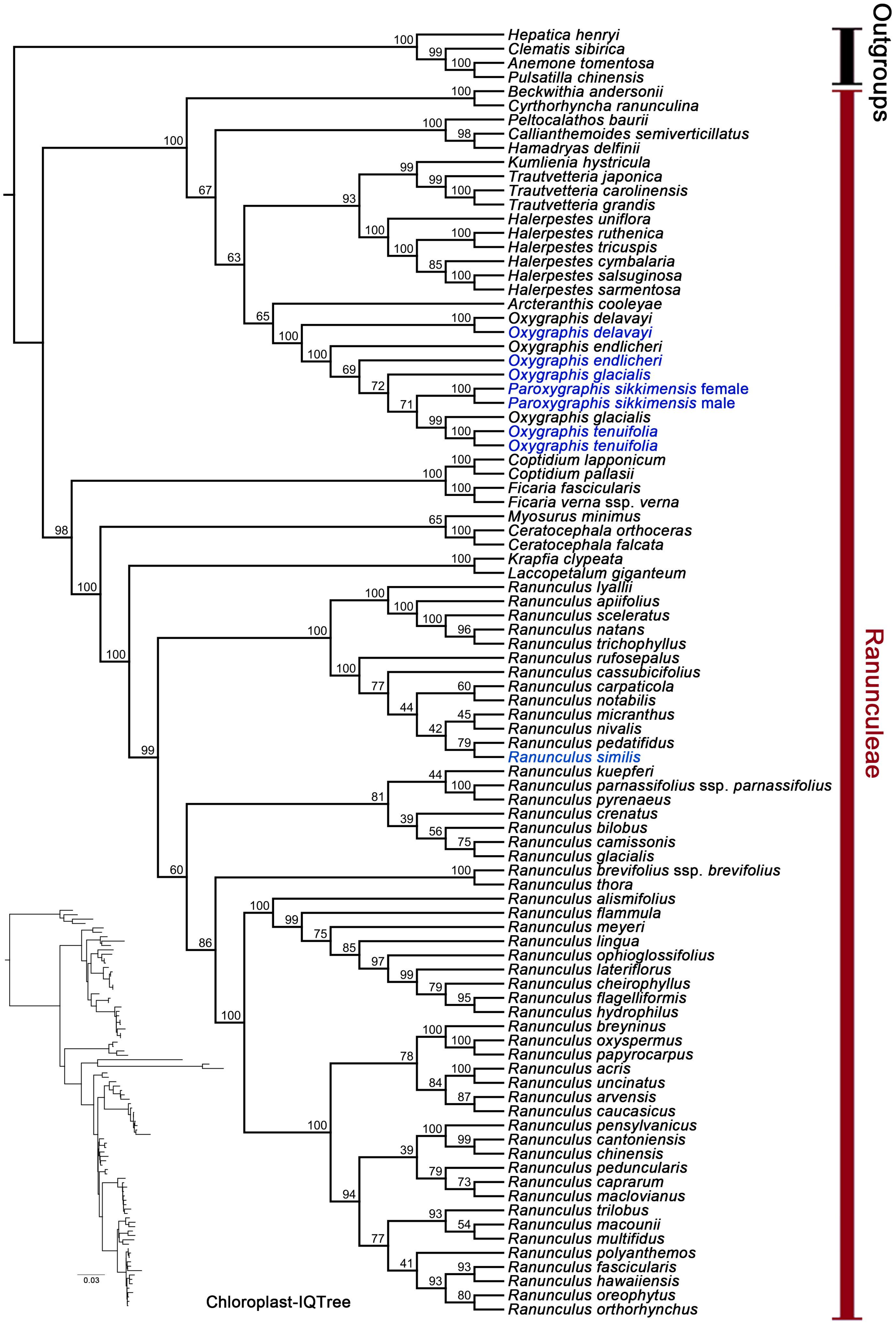

Outgroups
Ranunculeae
Hepatica henryi
Clematis sibirica
Anemone tomentosa
Pulsatilla chinensis
Beckwithia andersonii
Cyrthorhyncha ranunculina
Peltocalathos baurii
Callianthemoides semiverticillatus
Hamadryas delfinii
Kumlienia hystricula
Trautvetteria japonica
Trautvetteria carolinensis
Trautvetteria grandis
Halerpestes uniflora
Halerpestes ruthenica
Halerpestes tricuspis
Halerpestes cymbalaria
Halerpestes salsuginosa
Halerpestes sarmentosa
Arcteranthis cooleyae
Oxygraphis delavayi
Oxygraphis delavayi
Oxygraphis endlicheri
Oxygraphis endlicheri
Oxygraphis glacialis
Paroxygraphis sikkimensis female
Paroxygraphis sikkimensis male
Oxygraphis glacialis
Oxygraphis tenuifolia
Oxygraphis tenuifolia
Coptidium lapponicum
Coptidium pallasii
Ficaria fascicularis
Ficaria verna ssp. verna
Myosurus minimus
Ceratocephala orthoceras
Ceratocephala falcata
Krapfia clypeata
Laccopetalum giganteum
Ranunculus lyallii
Ranunculus apiifolius
Ranunculus sceleratus
Ranunculus natans
Ranunculus trichophyllus
Ranunculus rufosepalus
Ranunculus cassubicifolius
Ranunculus carpaticola
Ranunculus notabilis
Ranunculus micranthus
Ranunculus nivalis
Ranunculus pedatifidus
Ranunculus similis
Ranunculus kuepferi
Ranunculus parnassifolius ssp. parnassifolius
Ranunculus pyrenaeus
Ranunculus crenatus
Ranunculus bilobus
Ranunculus camissonis
Ranunculus glacialis
Ranunculus brevifolius ssp. brevifolius
Ranunculus thora
Ranunculus alismifolius
Ranunculus flammula
Ranunculus meyeri
Ranunculus lingua
Ranunculus ophioglossifolius
Ranunculus lateriflorus
Ranunculus cheirophyllus
Ranunculus flagelliformis
Ranunculus hydrophilus
Ranunculus breyninus
Ranunculus oxyspermus
Ranunculus papyrocarpus
Ranunculus acris
Ranunculus uncinatus
Ranunculus arvensis
Ranunculus caucasicus
Ranunculus pensylvanicus
Ranunculus cantoniensis
Ranunculus chinensis
Ranunculus peduncularis
Ranunculus caprarum
Ranunculus maclovianus
Ranunculus trilobus
Ranunculus macounii
Ranunculus multifidus
Ranunculus polyanthemos
Ranunculus fascicularis
Ranunculus hawaiiensis
Ranunculus oreophytus
Ranunculus orthorhynchus
0.03
Chloroplast-IQTree

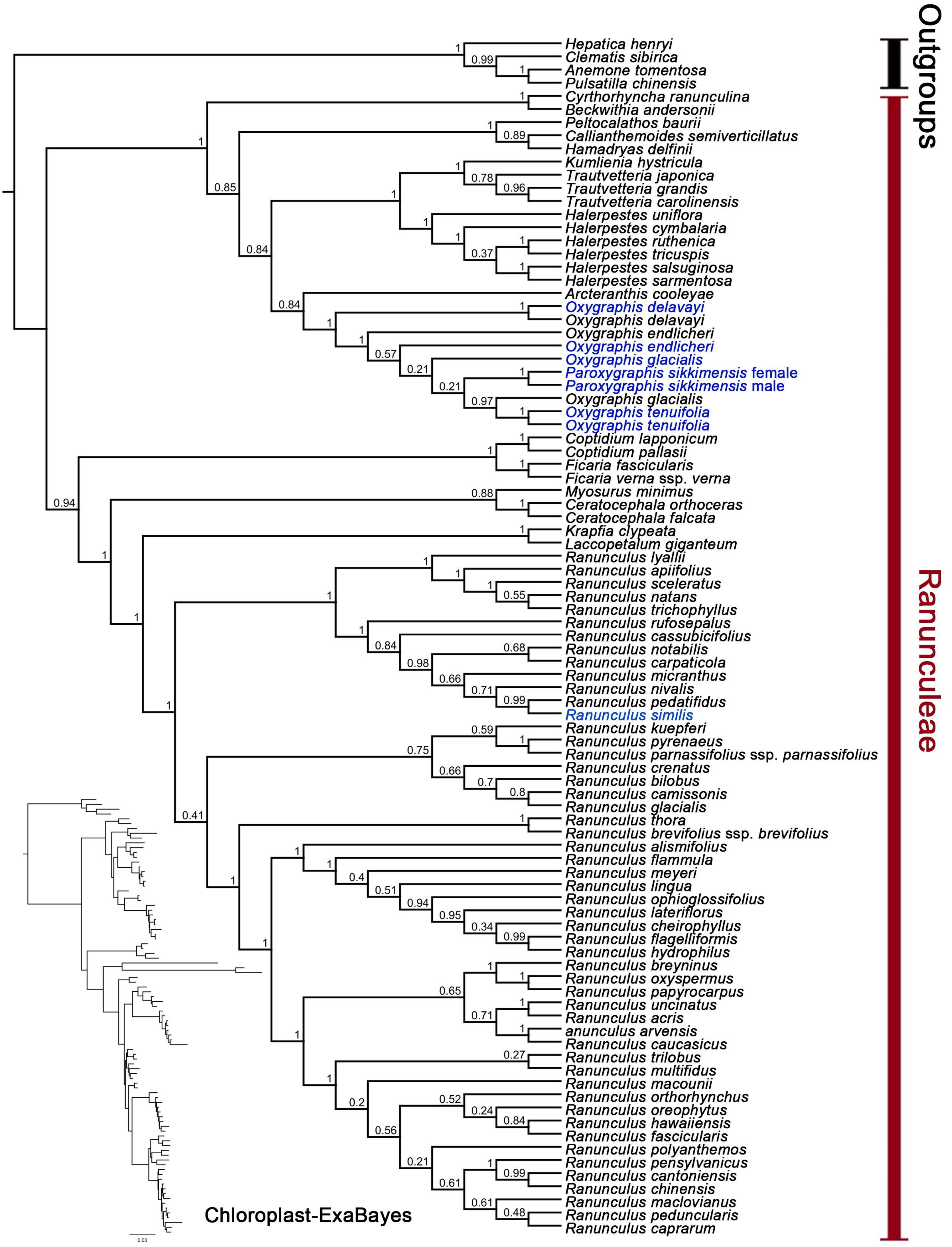

Outgroups
Ranunculeae
Hepatica henryi
Clematis sibirica
Anemone tomentosa
Pulsatilla chinensis
Cyrthorhyncha ranunculina
Beckwithia andersonii
Peltocalathos baurii
Callianthemoides semiverticillatus
Hamadryas delfinii
Kumlienia hystricula
Trautvetteria japonica
Trautvetteria grandis
Trautvetteria carolinensis
Halerpestes uniflora
Halerpestes cymbalaria
Halerpestes ruthenica
Halerpestes tricuspis
Halerpestes salsuginosa
Halerpestes sarmentosa
Arcteranthis cooleyae
Oxygraphis delavayi
Oxygraphis delavayi
Oxygraphis endlicheri
Oxygraphis endlicheri
Oxygraphis glacialis
Paroxygraphis sikkimensis female
Paroxygraphis sikkimensis male
Oxygraphis glacialis
Oxygraphis tenuifolia
Oxygraphis tenuifolia
Coptidium lapponicum
Coptidium pallasii
Ficaria fascicularis
Ficaria verna ssp. verna
Myosurus minimus
Ceratocephala orthoceras
Ceratocephala falcata
Krapfia clypeata
Laccopetalum giganteum
Ranunculus lyallii
Ranunculus apiifolius
Ranunculus sceleratus
Ranunculus natans
Ranunculus trichophyllus
Ranunculus rufosepalus
Ranunculus cassubicifolius
Ranunculus notabilis
Ranunculus carpaticola
Ranunculus micranthus
Ranunculus nivalis
Ranunculus pedatifidus
Ranunculus similis
Ranunculus kuepferi
Ranunculus pyrenaeus
Ranunculus parnassifolius ssp. parnassifolius
Ranunculus crenatus
Ranunculus bilobus
Ranunculus camissonis
Ranunculus glacialis
Ranunculus thora
Ranunculus brevifolius ssp. brevifolius
Ranunculus alismifolius
Ranunculus flammula
Ranunculus meyeri
Ranunculus lingua
Ranunculus ophioglossifolius
Ranunculus lateriflorus
Ranunculus cheirophyllus
Ranunculus flagelliformis
Ranunculus hydrophilus
Ranunculus breyninus
Ranunculus oxyspermus
Ranunculus papyrocarpus
Ranunculus uncinatus
Ranunculus acris
anunculus arvensis
Ranunculus caucasicus
Ranunculus trilobus
Ranunculus multifidus
Ranunculus macounii
Ranunculus orthorhynchus
Ranunculus oreophytus
Ranunculus hawaiiensis
Ranunculus fascicularis
Ranunculus polyanthemos
Ranunculus pensylvanicus
Ranunculus cantoniensis
Ranunculus chinensis
Ranunculus maclovianus
Ranunculus peduncularis
Ranunculus caprarum
0.03
Chloroplast-ExaBayes

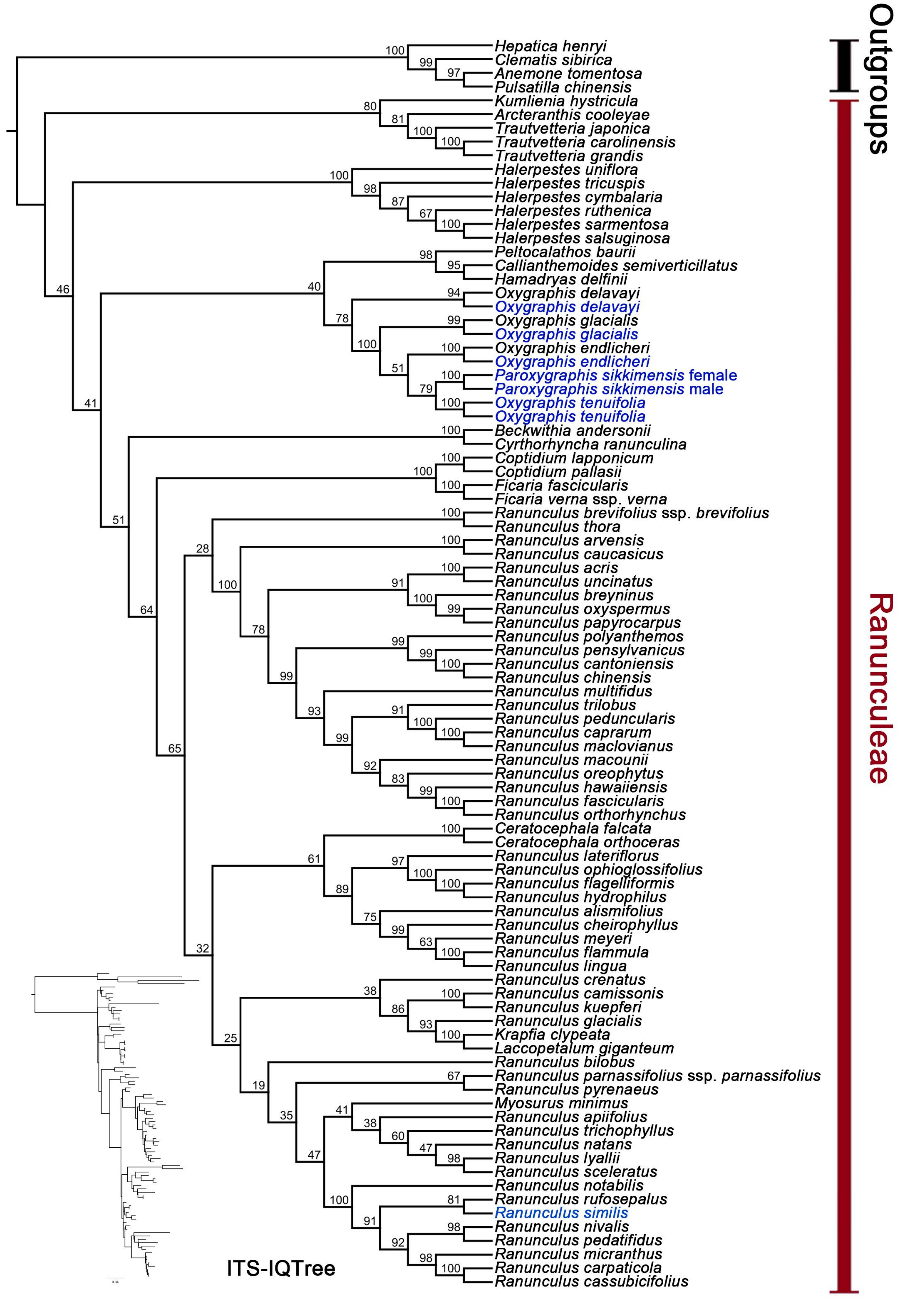

Outgroups
Ranunculeae
Hepatica henryi
Clematis sibirica
Anemone tomentosa
Pulsatilla chinensis
Kumlienia hystricula
Arcteranthis cooleyae
Trautvetteria japonica
Trautvetteria carolinensis
Trautvetteria grandis
Halerpestes uniflora
Halerpestes tricuspis
Halerpestes cymbalaria
Halerpestes ruthenica
Halerpestes sarmentosa
Halerpestes salsuginosa
Peltocalathos baurii
Callianthemoides semiverticillatus
Hamadryas delfinii
Oxygraphis delavayi
Oxygraphis delavayi
Oxygraphis glacialis
Oxygraphis glacialis
Oxygraphis endlicheri
Oxygraphis endlicheri
Paroxygraphis sikkimensis female
Paroxygraphis sikkimensis male
Oxygraphis tenuifolia
Oxygraphis tenuifolia
Beckwithia andersonii
Cyrthorhyncha ranunculina
Coptidium lapponicum
Coptidium pallasii
Ficaria fascicularis
Ficaria verna ssp. verna
Ranunculus brevifolius ssp. brevifolius
Ranunculus thora
Ranunculus arvensis
Ranunculus caucasicus
Ranunculus acris
Ranunculus uncinatus
Ranunculus breyninus
Ranunculus oxyspermus
Ranunculus papyrocarpus
Ranunculus polyanthemos
Ranunculus pensylvanicus
Ranunculus cantoniensis
Ranunculus chinensis
Ranunculus multifidus
Ranunculus trilobus
Ranunculus peduncularis
Ranunculus caprarum
Ranunculus maclovianus
Ranunculus macounii
Ranunculus oreophytus
Ranunculus hawaiiensis
Ranunculus fascicularis
Ranunculus orthorhynchus
Ceratocephala falcata
Ceratocephala orthoceras
Ranunculus lateriflorus
Ranunculus ophioglossifolius
Ranunculus flagelliformis
Ranunculus hydrophilus
Ranunculus alismifolius
Ranunculus cheirophyllus
Ranunculus meyeri
Ranunculus flammula
Ranunculus lingua
Ranunculus crenatus
Ranunculus camissonis
Ranunculus kuepferi
Ranunculus glacialis
Krapfia clypeata
Laccopetalum giganteum
Ranunculus bilobus
Ranunculus parnassifolius ssp. parnassifolius
Ranunculus pyrenaeus
Myosurus minimus
Ranunculus apiifolius
Ranunculus trichophyllus
Ranunculus natans
Ranunculus lyallii
Ranunculus sceleratus
Ranunculus notabilis
Ranunculus rufosepalus
Ranunculus similis
Ranunculus nivalis
Ranunculus pedatifidus
Ranunculus micranthus
Ranunculus carpaticola
Ranunculus cassubicifolius
ITS-IQTree

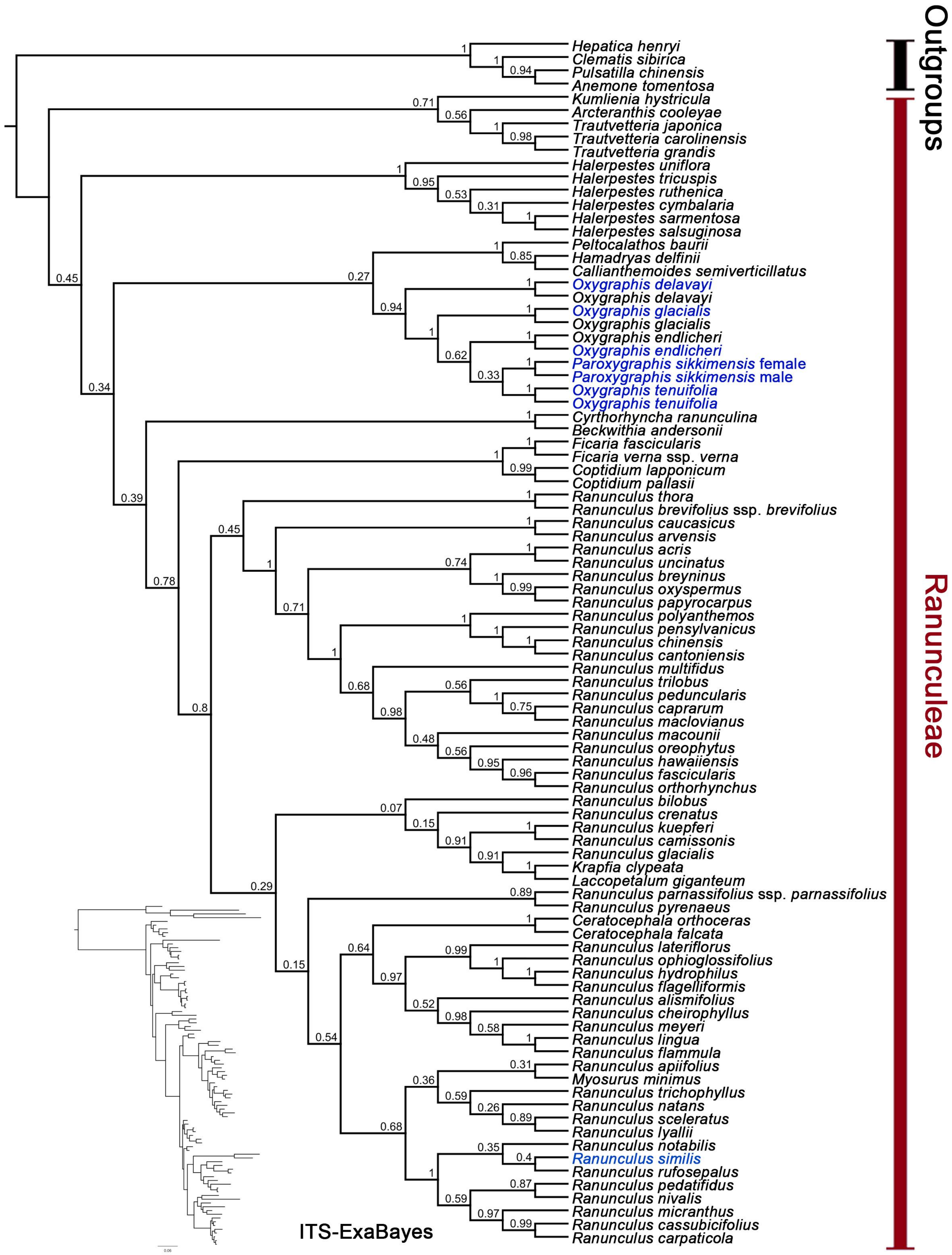

Outgroups
Ranunculeae
Hepatica henryi
Clematis sibirica
Pulsatilla chinensis
Anemone tomentosa
Kumlienia hystricula
Arcteranthis cooleyae
Trautvetteria japonica
Trautvetteria carolinensis
Trautvetteria grandis
Halerpestes uniflora
Halerpestes tricuspis
Halerpestes ruthenica
Halerpestes cymbalaria
Halerpestes sarmentosa
Halerpestes salsuginosa
Peltocalathos baurii
Hamadryas delfinii
Callianthemoides semiverticillatus
Oxygraphis delavayi
Oxygraphis delavayi
Oxygraphis glacialis
Oxygraphis glacialis
Oxygraphis endlicheri
Oxygraphis endlicheri
Paroxygraphis sikkimensis female
Paroxygraphis sikkimensis male
Oxygraphis tenuifolia
Oxygraphis tenuifolia
Cyrthorhyncha ranunculina
Beckwithia andersonii
Ficaria fascicularis
Ficaria verna ssp. verna
Coptidium lapponicum
Coptidium pallasii
Ranunculus thora
Ranunculus brevifolius ssp. brevifolius
Ranunculus caucasicus
Ranunculus arvensis
Ranunculus acris
Ranunculus uncinatus
Ranunculus breyninus
Ranunculus oxyspermus
Ranunculus papyrocarpus
Ranunculus polyanthemos
Ranunculus pensylvanicus
Ranunculus chinensis
Ranunculus cantoniensis
Ranunculus multifidus
Ranunculus trilobus
Ranunculus peduncularis
Ranunculus caprarum
Ranunculus maclovianus
Ranunculus macounii
Ranunculus oreophytus
Ranunculus hawaiiensis
Ranunculus fascicularis
Ranunculus orthorhynchus
Ranunculus bilobus
Ranunculus crenatus
Ranunculus kuepferi
Ranunculus camissonis
Ranunculus glacialis
Krapfia clypeata
Laccopetalum giganteum
Ranunculus parnassifolius ssp. parnassifolius
Ranunculus pyrenaeus
Ceratocephala orthoceras
Ceratocephala falcata
Ranunculus lateriflorus
Ranunculus ophioglossifolius
Ranunculus hydrophilus
Ranunculus flagelliformis
Ranunculus alismifolius
Ranunculus cheirophyllus
Ranunculus meyeri
Ranunculus lingua
Ranunculus flammula
Ranunculus apiifolius
Myosurus minimus
Ranunculus trichophyllus
Ranunculus natans
Ranunculus sceleratus
Ranunculus lyallii
Ranunculus notabilis
Ranunculus similis
Ranunculus rufosepalus
Ranunculus pedatifidus
Ranunculus nivalis
Ranunculus micranthus
Ranunculus cassubicifolius
Ranunculus carpaticola
ITS-ExaBayes
0.06
